# Modulation of Quorum Sensing and Virulence Gene Expression in *Escherichia coli* by Extracts of *Physalis alkekengi*

**DOI:** 10.1111/1758-2229.70371

**Published:** 2026-06-02

**Authors:** Mansoureh Esfandiyari Doolaby, Mohammad Javad Mehdipour Moghaddam, Akbar Norastehnia

**Affiliations:** ^1^ Department of Biology, Faculty of Science University of Guilan Rasht Iran

**Keywords:** antibacterial, *E. coli*, *Physalis alkekengi*, quorum sensing, real‐time PCR

## Abstract

The rise of multidrug‐resistant (MDR) bacteria underscores the need for new antimicrobials. In uropathogenic *Escherichia coli* , quorum sensing and adhesion play central roles in pathogenicity; therefore, *luxS* and *fimH* were selected as virulence targets. This study examined the antibacterial, antibiofilm and gene‐modulating effects of ethanolic and aqueous root extracts of *Physalis alkekengi* L. against three *E. coli* isolates (EC1, EC2, EC3). Phytochemical analysis confirmed the presence of phenolics, flavonoids, flavonols and anthocyanins, along with measurable antioxidant capacity. Antimicrobial activity was assessed using agar well diffusion, disk diffusion and broth microdilution on the MDR isolate EC2. Biofilm inhibition was quantified using a microtiter plate, and changes in *luxS* and *fimH* expression were evaluated by real‐time PCR. The ethanolic extract contained higher flavonoid and flavonol levels, whereas the aqueous extract was richer in phenolics. Anthocyanins and DPPH activity were undetectable in both extracts. The ethanolic extract completely inhibited EC3 at 10 mg/mL, although no MBC was observed for EC1, EC2 or any isolate exposed to the aqueous extract. In EC2, the ethanolic extract produced larger but variable inhibition zones, reduced biofilm by approximately 63% and significantly downregulated *luxS* and *fimH* (*p* < 0.05), supporting the potential of *P. alkekengi* as a natural antivirulence agent.

## Graphical abstract

Root extracts of *Physalis alkekengi* were examined for activity against multidrug‐resistant (MDR) *Escherichia coli* . The ethanolic extract suppressed bacterial growth, lowered biofilm formation by ~63%, and markedly reduced expression of *luxS* and *fimH*. Findings highlight *P. alkekengi* as a potential natural strategy for targeting virulence in MDR urinary pathogens.

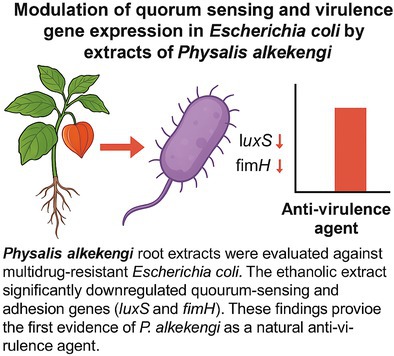

## 1 Introduction

The rapid rise of multidrug‐resistant (MDR) bacteria has become a major global health challenge, threatening the effectiveness of existing antibiotics and driving the search for new antimicrobial approaches (Ventola 2015; World Health Organization 2020). Natural products, particularly medicinal plants, represent an important source of structurally diverse bioactive molecules with broad pharmacological potential. For centuries, they have played a central role in traditional medical practices and continue to provide valuable scaffolds for modern drug discovery efforts (Newman and Cragg 2020; McCormick and McBride 2015). As antimicrobial resistance (AMR) becomes increasingly widespread, the exploration of plant‐derived compounds as alternative therapeutic candidates is gaining renewed attention (Alzohairy 2016).

*Physalis alkekengi* L. (bladder cherry), a member of the Solanaceae family, is widely recognised in traditional medicine systems. Ethnobotanical literature reports that its fruits and calyces are commonly prepared as infusions, decoctions or extracts, primarily to relieve respiratory ailments (such as cough, pharyngitis and mucus accumulation), urinary tract symptoms (e.g., dysuria) and various inflammatory or infectious conditions. Phytochemical analyses have revealed the presence of numerous constituents, including physalins, flavonoids, alkaloids and phenolic compounds. Both crude extracts and isolated metabolites from the plant have shown antimicrobial and anti‐inflammatory effects in vitro and in vivo (Tundis et al. 2014; Alzohairy 2016; Wang et al. 2023; Zuberi and Hussain 2017). Although most previous investigations have focused on the fruits and calyces, other plant parts—such as the roots—remain comparatively underexplored. In this study, ethanolic and aqueous extracts of *P. alkekengi* roots were prepared (as detailed in the Methods section) and examined for their antimicrobial and antivirulence activities against clinical uropathogenic isolates.

*Escherichia coli* remains one of the most significant Gram‐negative bacterial pathogens, responsible for numerous human infections. Uropathogenic *E. coli* (UPEC) is the predominant cause of urinary tract infections (UTIs), which are among the most prevalent bacterial infections globally (Flores‐Mireles et al. 2015). Multiple virulence determinants, including adhesins, toxins and biofilm formation, mediate UPEC pathogenicity. Among these factors, the fimH gene, which encodes the type 1 fimbrial adhesin, is essential for adherence to uroepithelial cells and for establishing infection (Klemm and Schembri 2000).

Quorum sensing (QS) enables bacteria to regulate gene expression in response to cell density. In *E. coli* , the AI‐2/LuxS signalling pathway is the best characterised and controls motility, biofilm formation and virulence‐related genes (Xavier and Bassler 2003; Rutherford and Bassler 2012). Additional systems, such as the AI‐3/QseC–QseB signalling network, integrate bacterial communication with host hormones, including epinephrine and norepinephrine, influencing motility and pathogenic behaviour (Sperandio et al. 2003; Walters and Sperandio 2006). Indole‐mediated signalling, derived from tryptophan metabolism, also contributes to stress adaptation and biofilm regulation (Lee and Lee 2010). Although *E. coli* does not synthesise acyl‐homoserine lactones (AHLs), it can detect AHLs produced by neighbouring microbes, enabling interspecies communication. Collectively, these interconnected systems regulate virulence determinants such as FimH and LuxS and represent promising targets for antivirulence interventions.

Beyond their clinical importance, QS and biofilm‐associated behaviours play fundamental roles in shaping microbial survival and persistence in natural and host‐associated environments. These communication systems enable bacteria to coordinate collective responses to population density and environmental signals, thereby influencing microbial colonisation, competition and ecological fitness (Ng and Bassler 2009; Papenfort and Bassler 2016). Increasing evidence also indicates that plant‐derived secondary metabolites can interfere with QS pathways and attenuate virulence without necessarily inhibiting bacterial growth, highlighting their ecological and therapeutic relevance (Vattem et al. 2007; Kalia 2013). Therefore, investigating the effects of *P. alkekengi* root extracts on QS–related genes, such as *luxS*, and virulence determinants, such as *fimH*, may provide insights into environmentally relevant mechanisms that regulate bacterial communication, adaptation and pathogenic potential.

Given this background, the present study aimed to investigate the antimicrobial, antibiofilm and gene‐modulatory effects of ethanolic and aqueous extracts of *P. alkekengi* roots against clinical UPEC isolates. Specifically, we evaluated the influence of these extracts on the expression of *fimH* and *luxS* to assess the potential of this medicinal plant as a natural therapeutic candidate for UTI management.

## 2 Material and Methods

### 2.1 Plant Sample and Preparation of Extracts

Root samples of *P. alkekengi* L. were obtained during the summer of 2023 from the Manjil area in Guilan Province, Iran (36°45′5″ N, 43°23′28″ E). The plant identity was confirmed by a botanist from the Department of Biology at the University of Guilan, and a voucher specimen was archived in the departmental herbarium. The collected roots were rinsed thoroughly with distilled water to eliminate soil residues, air‐dried in the shade at ambient temperature for approximately 2 weeks, and subsequently ground into a fine powder using an electric mill.

Ethanolic and aqueous extracts were prepared according to a standard maceration protocol (Harborne 1998; Edeoga et al. 2005). In brief, 5 g of the dried root powder was combined with 50 mL of the extraction solvent and kept in a shaker incubator at 25°C with gentle agitation (100 rpm) for 72 h. An 80% ethanol solution served as the solvent for the ethanolic extract, while sterile distilled water was used for the aqueous extract. After the incubation period, the mixtures were centrifuged at 8000 rpm for 15 min at 24°C, and the resulting supernatants were transferred into fresh tubes. These solutions were dried in an oven at 40°C–50°C until a semi‐solid crude extract was obtained. Approximately 0.02 g of the dried material was dissolved in 5% DMSO. Before use, extracts were sterilised through 0.45 μm membrane filters for antimicrobial experiments and 0.22 μm filters for molecular analyses. The filtrates were concentrated using a rotary evaporator and stored at 4°C until further experiments.

A stock solution at 20 mg/mL was prepared by dissolving 0.02 g of crude extract in 1 mL of 5% DMSO (ethanolic extract) or sterile distilled water (aqueous extract), followed by vigorous mixing. From this stock, two‐fold serial dilutions were prepared to yield final concentrations of 10, 5, 2.5 and 1.25 mg/mL.

### 2.2 Phytochemical Analysis

The phytochemical content of the extracts was assessed as follows:

#### 2.2.1 Total Phenolic Content (TPC)

TPC of the plant extracts was quantified using the Folin–Ciocalteu colorimetric assay (Singleton and Rossi 1965). In this procedure, 100 μL of the extract was combined with an equal volume of 1% Folin–Ciocalteu reagent and incubated at room temperature for 5 min. Subsequently, 2 mL of a 2% sodium carbonate solution was added, and the reaction mixture was incubated in the dark for 30 min. The absorbance of the resulting solution was then recorded at 720 nm using a UV–Vis spectrophotometer. Gallic acid served as the calibration standard, and the phenolic content of the samples was reported as milligrams of gallic acid equivalents (GAE) per gram of dry extract.

#### 2.2.2 Total Flavonoids

The concentration of flavonoid compounds in the extracts was determined following the aluminium chloride colorimetric procedure described by Chang et al. (2002). For this assay, 500 μL of the extract was combined with 1.5 mL of 80% ethanol, followed by the addition of 100 μL of 10% aluminium chloride solution, 100 μL of 1 M potassium acetate and 2.8 mL of distilled water. The mixture was allowed to stand at room temperature for 30 min, after which its absorbance was measured at 450 nm. Quercetin was used to generate the standard calibration curve, and the results were expressed as milligrams of quercetin equivalents (QE) per gram of dried extract.

#### 2.2.3 Flavonols

The flavonol content in the extracts was assessed using the spectrophotometric procedure described by Pękal and Pyrzynska (2014). In this assay, 1 mL of the extract was combined with 2 mL of a 2% aluminium chloride solution and 6 mL of 5% sodium acetate. The reaction mixture was then kept at room temperature for 2.5 h to allow complex formation. After incubation, the absorbance was recorded at 440 nm. Quercetin was used to generate the standard calibration curve, and the values were expressed as milligrams of QE per gram of dried extract.

#### 2.2.4 Anthocyanins

The concentration of anthocyanins in the extracts was evaluated using a spectrophotometric method adapted from those described by Giusti and Wrolstad (2001) and Hosseinian et al. (2008), with minor procedural adjustments. To prepare the samples, 0.05 g of dried plant powder was dispersed in 9.9 mL of 96% ethanol for the ethanolic extract, or in distilled water for the aqueous extract. Subsequently, 0.1 mL of HCl was added, and the mixture was vortex‐mixed for 30 s. The samples were then kept at −20°C for 24 h to enhance pigment extraction. After thawing, the suspensions were centrifuged at 12,000 rpm for 15 min at 4°C, and the resulting supernatants were transferred to clean tubes. The absorbance of each sample was recorded at 550 nm using a UV–Vis spectrophotometer, with the corresponding reagent mixture (without plant material) serving as the blank. Anthocyanin content was calculated using the formula A = εLC, where A represents the measured absorbance, ε the molar absorptivity coefficient, L the optical path length and C the concentration of the solution.

#### 2.2.5 Antioxidant Activity

The antioxidant capacity of the extracts was assessed using the DPPH free‐radical scavenging assay described by Brand‐Williams et al. (1995). For this analysis, a 0.1 mM DPPH solution was freshly prepared in methanol. Then, 950 μL of the DPPH solution was combined with 50 μL of the extract at various concentrations. The mixture was vortexed briefly, then incubated in the dark at room temperature for 30 min. After incubation, the absorbance was measured at 517 nm.

The percentage of radical scavenging activity was calculated using the equation:

$$\%\text{Inhibition}=\left(\left(A\_\text{control}-A\_\text{sample}\right)/A\_\text{control}\right)\times 100$$

The IC_50_ value, representing the extract concentration required to quench 50% of DPPH radicals, was obtained from the plotted dose–response curve.

### 2.3 Bacterial Samples

Clinical isolates of *E. coli* were obtained from the Razi Pathobiology Laboratory in Rasht, Iran. The isolates were initially characterised using standard biochemical tests, and their identities were subsequently confirmed through molecular methods. All three *E. coli* strains originated solely from urine samples and were selected for inclusion in this study.

### 2.4 Antimicrobial Assay

#### 2.4.1 Well Diffusion Test

A bacterial suspension was prepared and adjusted to approximately 1 × 10^6^ CFU/mL, then spread evenly across Mueller–Hinton agar (MHA) plates to obtain a uniform lawn. Wells with a diameter of 6 mm were aseptically punched into the agar, and each well was first filled with 15 μL of molten nutrient agar to prevent sample leakage. Afterward, 30 μL of the aqueous or ethanolic extracts at concentrations of 10, 5, 2.5 and 1.25 mg/mL were added to the wells in triplicate. The plates were incubated at 37°C for 24 h, and the inhibition zone diameters were subsequently recorded in millimetres, following the method described by Balouiri et al. (2016).

#### 2.4.2 Broth Microdilution Method

The minimum inhibitory concentration (MIC) and minimum bactericidal concentration (MBC) of the extracts were determined using the broth microdilution method, following the CLSI recommendations with slight adjustments (CLSI 2023). Fresh suspensions of the *E. coli* isolates were prepared from overnight growth on MHA and standardised to approximately 1 × 10^6^ CFU/mL. Two‐fold serial dilutions of each extract were prepared in Mueller–Hinton Broth (MHB) to obtain final concentrations of 10, 5, 2.5 and 1.25 mg/mL. For each test well, 100 μL of the bacterial suspension was added to 100 μL of the corresponding extract dilution. All experiments were performed in triplicate.

Appropriate controls were included: growth control (MHB plus bacterial inoculum), sterility control (MHB only), solvent control (5% DMSO or sterile distilled water), and positive control (bacterial suspension in MHB supplemented with gentamicin) (CLSI 2023; Wiegand et al. 2008). The microplates were then incubated for 24 h at 37°C without agitation. Bacterial proliferation was assessed by measuring optical density at 570 nm using a microplate reader. The MIC was defined as the lowest concentration of the extract that completely inhibited visible growth compared to the growth control.

To determine the MBC, 10 μL from wells lacking visible growth were streaked onto fresh MHA plates and incubated at 37°C for 24 h. The MBC was recorded as the lowest extract concentration producing no colony formation, corresponding to at least a 99.9% reduction in the initial bacterial load.

#### 2.4.3 Minimum Biofilm Inhibitory Concentration Assay (MBIC)

The MBIC of the ethanolic extracts was evaluated using a microtiter plate‐based biofilm formation assay with minor procedural modifications. Fresh cultures of the *E. coli* isolates grown overnight on MHA were suspended in sterile medium and adjusted to approximately 1 × 10^6^ CFU/mL. Serial two‐fold dilutions of the extracts (10, 5, 2.5 and 1.25 mg/mL) were prepared in MHB. In sterile 96‐well microplates, 100 μL of each extract dilution was mixed with an equal volume (100 μL) of the standardised bacterial suspension. Growth, sterility, solvent (5% DMSO) and positive controls were included as previously reported (Stepanović et al. 2007; Toole 2011).

The plates were incubated at 37°C without agitation for 48 h to allow biofilm formation. After incubation, nonadherent cells were removed by gently rinsing each well two to three times with sterile distilled water. The remaining biofilms were fixed and stained by adding 150 μL of 1% crystal violet solution and incubating for 15 min at room temperature. Excess stain was washed away with distilled water, and the plates were left inverted to air‐dry for 1–2 h. The retained crystal violet was then solubilised with 150 μL of 30% acetic acid for 15 min, and the absorbance was recorded at 570 nm using a microplate reader. The MBIC was defined as the lowest extract concentration that produced a significant reduction in biofilm biomass relative to the untreated control (Stepanović et al. 2007; Toole 2011).

### 2.5 Molecular Analyses

#### 2.5.1 Polymerase Chain Reaction (PCR)

Genomic DNA of the *E. coli* isolates was extracted using a simple boiling lysis procedure. In brief, overnight bacterial cultures were centrifuged, and the resulting pellets were resuspended in sterile buffer. The suspensions were heated at 95°C for 10 min, rapidly cooled on ice, and centrifuged once more to pellet cellular debris. The supernatant, containing crude genomic DNA, was collected and used directly as the PCR template.

PCR amplification targeted three genetic markers—*16S rRNA*, *luxS* and *fimH*—using gene‐specific primers listed in Table 1 (Zhu and Mekalanos 2003; Sheikh et al. 2001). The *16S rRNA* gene served as a housekeeping internal control, *luxS* represented a quorum‐sensing–associated gene, and *fimH* corresponded to an adhesion factor linked with virulence.

**TABLE 1:** Primer sequences and associated characteristics used for the amplification of target genes.

Target gene	Direction	Sequence (5′→3′)	Size (bp)	Tm (°C)	GC content
*16 rRNA*	Forward	TTACGACCAGGGCTACACAC	144	59.35	55
*16 rRNA*	Reverse	ACGATTACTAGCGATTCCGAC		57.87	47.62
*luxS*	Forward	AATCACCGTGTTCGATCTGC	220	57.30	50
*luxS*	Reverse	GCTCATCTGGCGTACCAATC		59.35	55
*fimH*	Forward	GCTGTGATGTTTCTGCTCGT	168	57.30	50
*fimH*	Reverse	AAAACGAGGCGGTATTGGTG		57.30	50

Amplifications were performed in a programmed thermal cycler under the following conditions: initial denaturation at 95°C for 15 min; then 45 cycles consisting of denaturation at 95°C for 30 s, annealing at 60°C for 30 s and extension at 72°C for 30 s. A final elongation step was executed at 72°C for 10 min to ensure complete synthesis. PCR products were then kept at 4°C until subsequent electrophoretic or sequencing analyses (Zhu and Mekalanos 2003; Sheikh et al. 2001).

#### 2.5.2 Quantification of *luxS* and *fimH* Genes Based on Quantitative Real‐Time PCR (qPCR)

Total RNA was extracted from EC2 cultures—either untreated or exposed to the plant extracts—using the RNX‐Plus reagent (CinnaGen, Iran) following the manufacturer's protocol. After 24 h of incubation, bacterial cells were collected by centrifugation (8000*g*, 12 min, 4°C). The resulting RNA pellets were dissolved in RNase‐free water and stored at −70°C. RNA concentration and purity were assessed using a NanoDrop spectrophotometer (Thermo Fisher Scientific, USA), and only samples with A260/280 ratios of 1.8–2.0 were selected for downstream analysis.

Complementary DNA (cDNA) synthesis was performed from 1 μg of total RNA using the RevertAid First Strand cDNA Synthesis Kit (Thermo Fisher Scientific, USA) with random hexamer primers in a final volume of 20 μL.

qPCR was conducted on a LightCycler 96 system (Roche, Switzerland) in 25 μL reaction mixtures containing 12.5 μL of 2× SYBR Green PCR Master Mix, 1 μL of each primer (10 μM), 2 μL of cDNA template and 8.5 μL of nuclease‐free water. The thermocycling conditions were as follows: initial denaturation at 95°C for 15 min, followed by 45 cycles of 95°C for 30 s, 60°C for 30 s and 72°C for 30 s, with a final extension at 72°C for 10 min. Melt curve analysis (65°C–95°C) was performed to confirm amplification specificity (Table 2).

**TABLE 2:** Thermal cycling conditions for qPCR analysis.

Stage	Temperature (°C)	Time (min)
Initial denaturation	95	15 min
Denaturation	95	30 s
Annealing	60	30 s
Extension	72	30 s

Relative transcript levels of *16S rRNA*, *luxS* and *fimH* were quantified, using *16S rRNA* as the endogenous reference gene. Primer pairs were designed using Primer‐BLAST (NCBI) and validated for specificity and acceptable amplification efficiency (90%–110%). Gene expression changes were calculated using the 2^−ΔΔC*t*^ method (Livak and Schmittgen 2001). All reactions were carried out in triplicate, and no‐template controls were included as quality assurance measures.

### 2.6 Statistical Analysis

Statistical analyses were carried out using GraphPad Prism version 9.0 (GraphPad Software, USA). Data were analysed by one‐way ANOVA followed by Tukey's post hoc multiple comparison test to determine differences among groups. A *p* value of less than 0.05 was considered indicative of statistical significance.

## 3 Results

**FIGURE 1:**
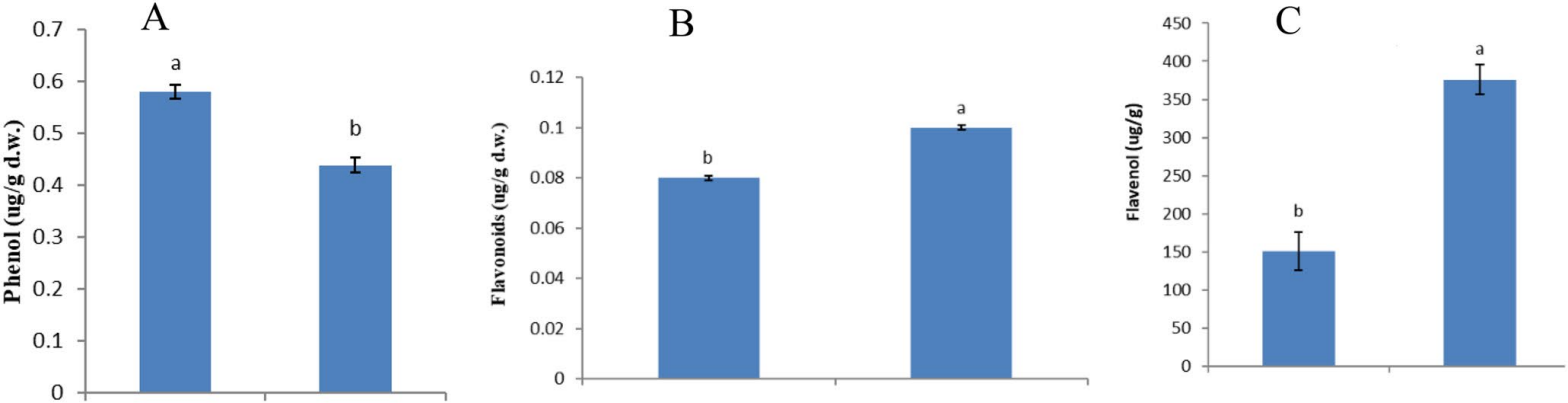
Levels of major phytochemicals, including phenols (A), flavonoids (B) and flavonols (C), in aqueous (aq) and ethanolic (et) extracts of *Physalis alkekengi* root.

A comparative analysis of secondary metabolites in aqueous and ethanolic extracts of *P. alkekengi* showed clear differences in their chemical composition. The aqueous extract contained significantly higher levels of phenolic compounds (0.58 ± 0.02 μg/g d.w.) than the ethanolic extract (0.45 ± 0.01 μg/g d.w.) (*p* < 0.05) (Figure 1A). Conversely, the ethanolic extract contained higher levels of flavonoids (0.11 ± 0.003 μg/g d.w.), whereas flavonol content was substantially greater in the aqueous extract (150 ± 10 μg/g d.w.) compared with the ethanolic extract (38 ± 15 μg/g d.w.) (*p* < 0.01). (Figure 1B,C). Anthocyanins and antioxidant activity (DPPH assay) were below detection limits and therefore omitted.

**FIGURE 2:**
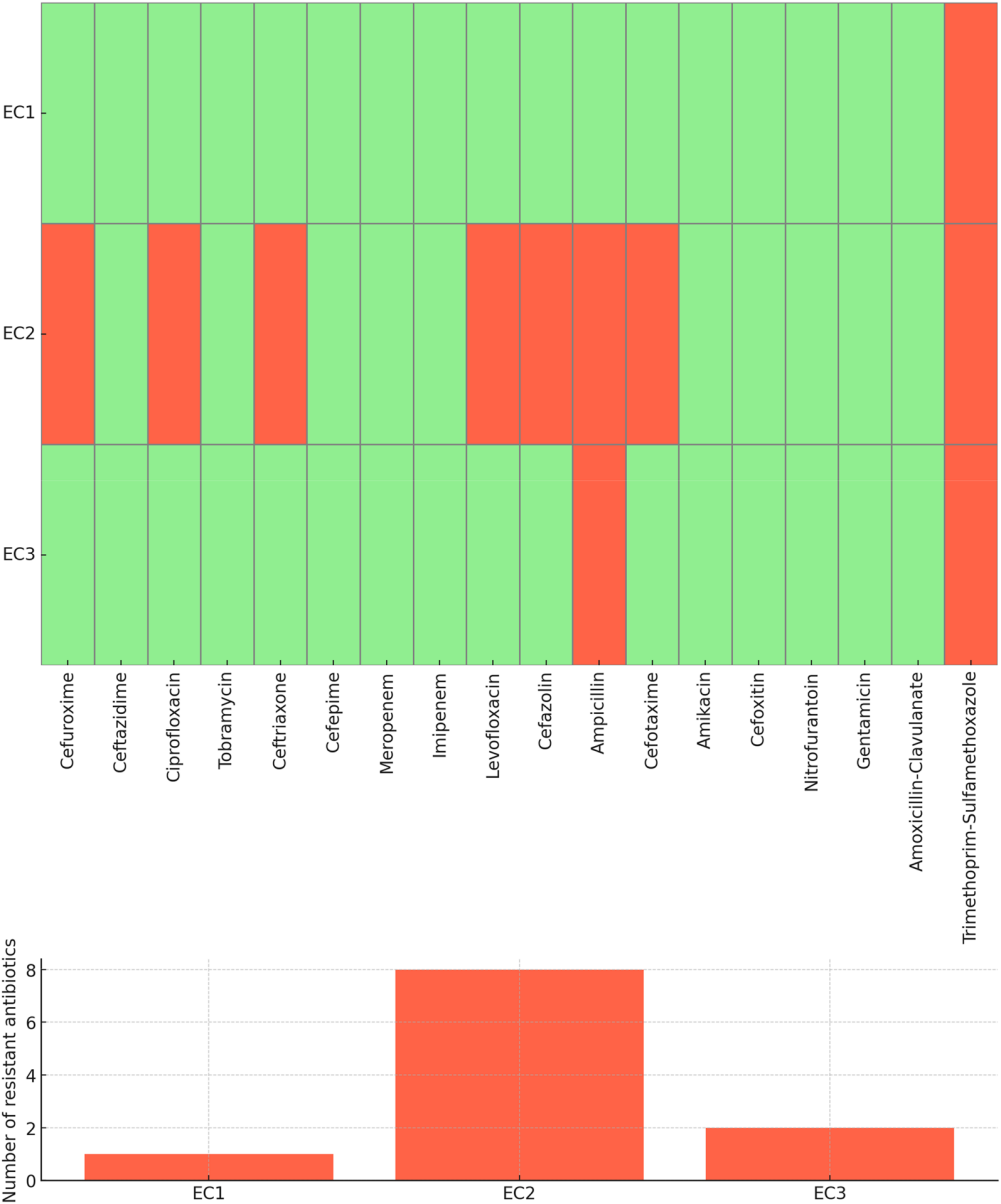
Antimicrobial response patterns of *Escherichia coli* isolates. Sensitivity (green) and resistance (red) across the evaluated antibiotics are visualised in the heatmap. The bar graph below shows the number of antibiotics to which each isolate is resistant, with EC2 demonstrating MDR.

Antibiotic susceptibility profiling of the three *E. coli* isolates revealed notable variability in resistance behaviour (Figure 2). EC1 exhibited resistance solely to cotrimoxazole, whereas EC3 showed resistance to both ampicillin and cotrimoxazole. EC2 displayed the broadest resistance pattern, being nonsusceptible to seven antibiotics, and was classified as MDR. All isolates were resistant to cotrimoxazole, indicating a shared resistance determinant. Despite these differences, carbapenems (imipenem and meropenem) and aminoglycosides (gentamicin, amikacin and tobramycin) remained effective against all three isolates, suggesting that these drug classes retain therapeutic potential for infections caused by these strains.

**FIGURE 3:**
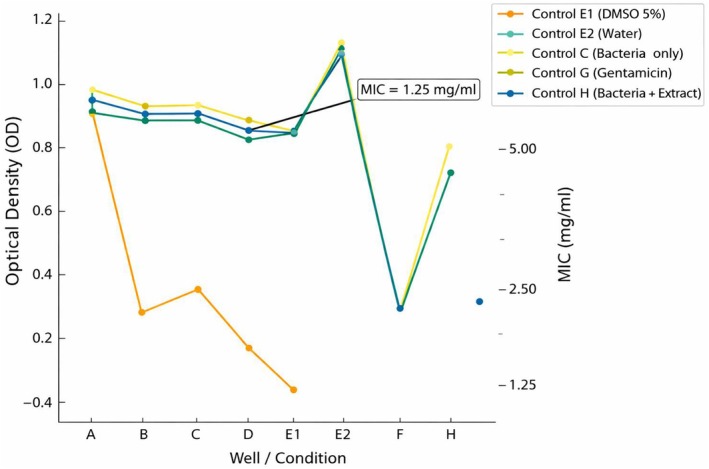
Comparison of optical density changes and MIC of *Physalis alkekengi* extract in different *Escherichia coli* strains. OD responses from the experimental and control setups are displayed, with MIC values (mg/mL) plotted on the secondary axis. The arrow marks the concentration identified as the MIC.

MIC assays showed that the ethanolic extract possessed greater antibacterial potency than the aqueous extract. For EC1, partial inhibition was observed at 10 mg/mL, while EC2 demonstrated limited inhibition at lower extract concentrations (Figure 3). EC3 was the most susceptible strain, showing complete inhibition at 10 mg/mL for both extracts, representing the lowest MIC among the isolates. The aqueous extract failed to achieve complete inhibition at any concentration tested, although EC3 showed the greatest reduction in growth. These findings underscore the differential susceptibility among the isolates, with EC3 being especially responsive to the plant extracts.

MBC determinations indicated that only EC3 showed complete killing at 10 mg/mL of the ethanolic extract, indicating that its MBC matched its MIC. MBC values could not be established for EC1 or EC2 with the ethanolic extract, nor for any strain treated with the aqueous extract, as none reached total inhibition at the tested concentrations.

**FIGURE 4:**
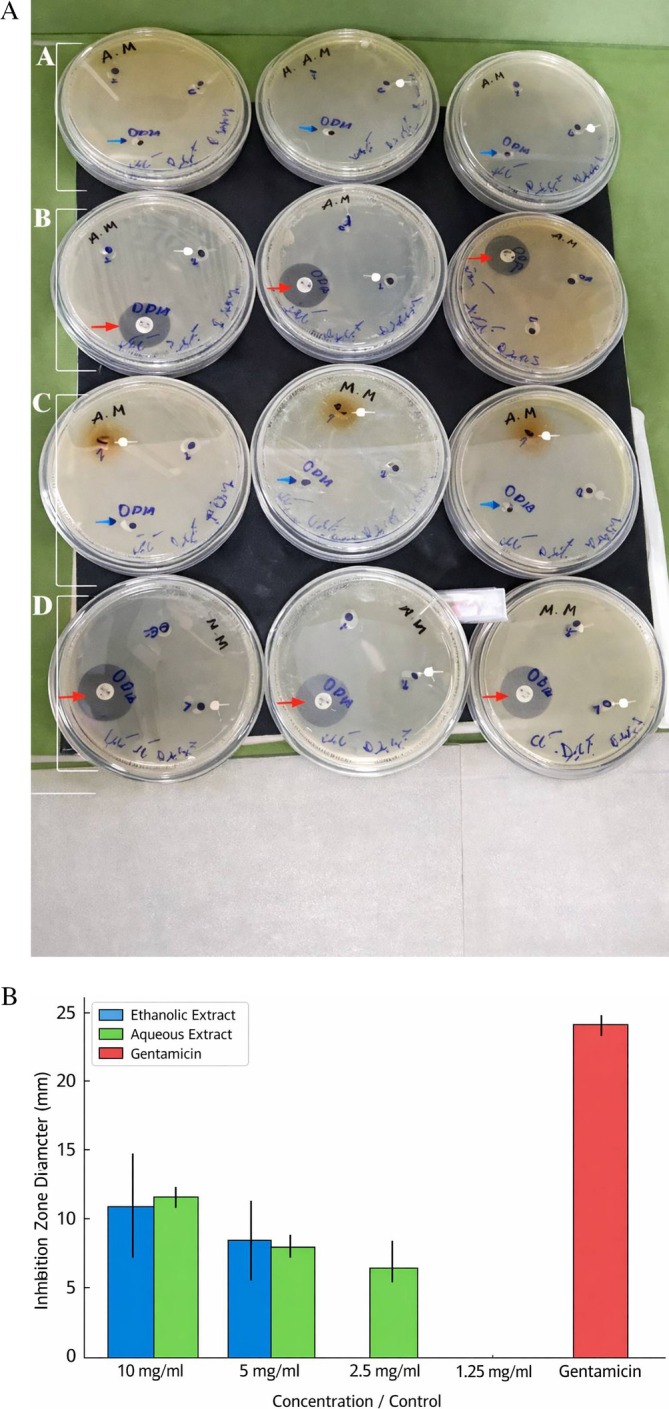
Evaluation of the antibacterial potential of ethanolic and aqueous *Physalis alkekengi* extracts against EC2 using the well diffusion method. (A) Example agar plates illustrating inhibition zones produced by each extract, alongside gentamicin (positive control) and DMSO (negative control). Rows represent: (A) ethanolic extract with DMSO; (B) ethanolic extract with gentamicin; (C) aqueous extract with DMSO and (D), aqueous extract with gentamicin. White arrows mark extract‐derived inhibition zones, blue arrows show the noninhibitory DMSO control, and red arrows indicate gentamicin activity. (B) Bar plot comparing inhibition‐zone diameters across extract concentrations and gentamicin.

Agar well diffusion assays, performed exclusively on EC2 as the MDR representative, further demonstrated the antibacterial activity of the extracts (Figure 4A–B). At 10 mg/mL, the ethanolic extract produced inhibition zones of 8–16 mm (mean 10.7 ± 3.8 mm), while the aqueous extract produced consistent zones of 12.0 ± 0.0 mm. At 5 mg/mL, inhibition decreased to 8.3 ± 2.4 mm for the ethanolic extract and remained at 8.0 ± 0.0 mm for the aqueous extract. At 2.5 mg/mL, the aqueous extract retained slight activity (6–8 mm), whereas the ethanolic extract showed no detectable inhibition; no activity was recorded for either extract at 1.25 mg/mL. The positive control, gentamicin, consistently produced significantly larger zones (23.7 ± 0.6 mm; *p* < 0.05). These outcomes demonstrate a concentration‐dependent response for the ethanolic extract, with greater variability, while the aqueous extract showed more stable, but generally moderate, activity.

Comparison of the broth microdilution and diffusion assays highlighted complementary rather than directly comparable patterns. In the MIC assay performed on EC1, EC2 and EC3, the ethanolic extract showed greater potency, with EC3 exhibiting complete inhibition at 10 mg/mL. In contrast, the aqueous extract failed to reach complete inhibition in any isolate. In the diffusion assay applied only to EC2, both extracts produced measurable activity at higher concentrations, although their inhibition profiles differed across concentrations. Overall, while EC3 was the most sensitive strain in MIC testing, EC2 exhibited the broadest susceptibility pattern when combining data from both assays, justifying its selection for subsequent molecular characterisation due to its clinical relevance and MDR profile.

**FIGURE 5:**
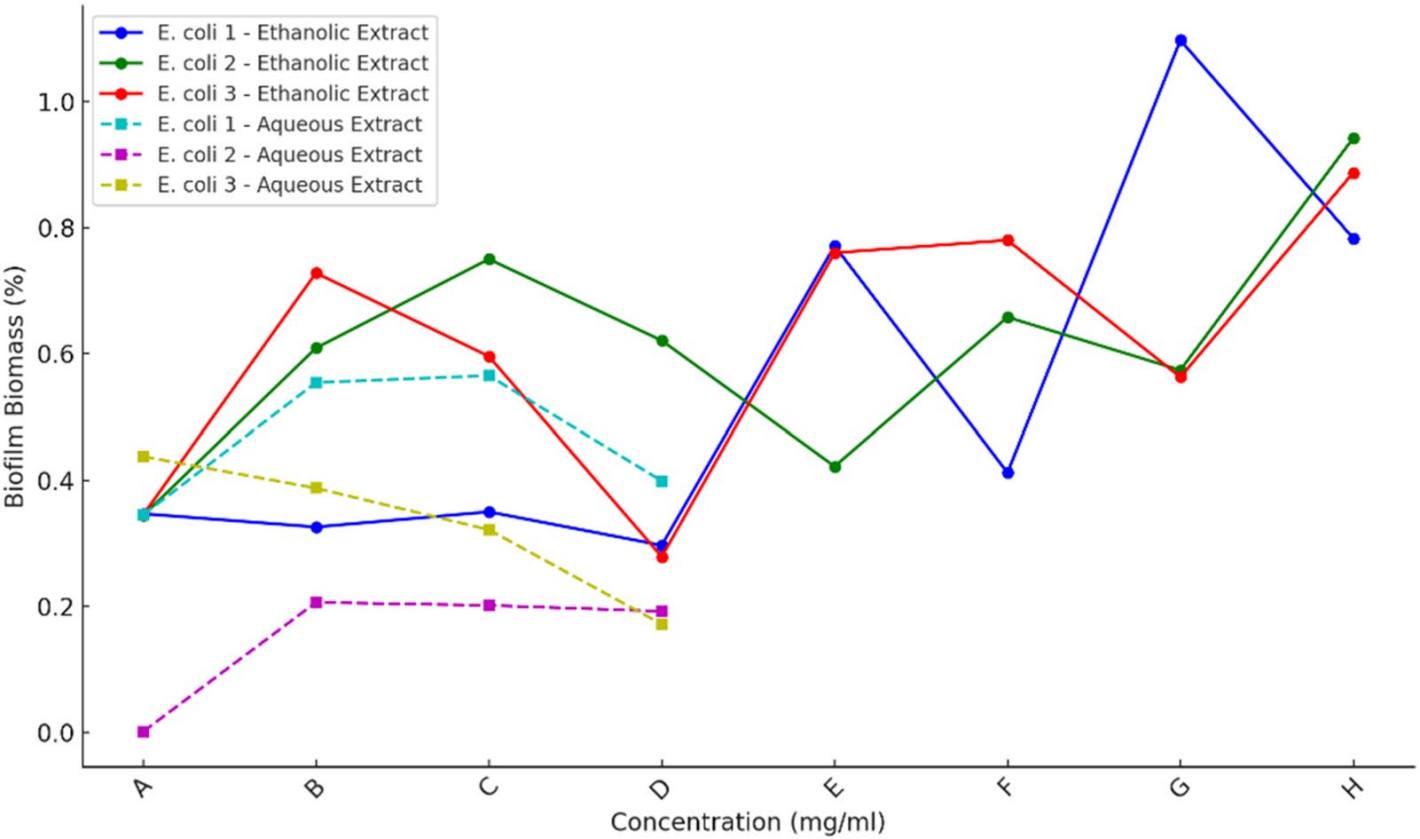
Evaluation of the antibiofilm potential of *Physalis alkekengi* ethanolic extract against *Escherichia coli* strains. Panels A–H indicate the following conditions: A: 10 mg/mL extract; B: 5 mg/mL extract; C: 2.5 mg/mL extract; D: 1.25 mg/mL extract; E: Medium with bacteria; F: Medium with bacteria plus 5% DMSO; G: Medium only; H: Medium with extract.

Biofilm inhibition assays revealed that both extracts affected biofilm biomass in a concentration‐dependent manner (Figure 5). The ethanolic extract consistently reduced biofilm formation across all isolates and concentrations. The strongest reduction occurred at 10 mg/mL, where EC3 showed a biomass of 0.345 ± 0.002, corresponding to a 63.5% decrease relative to the untreated control (Table 3). Even at lower concentrations (5 and 1.25 mg/mL), reductions of approximately 55% and 48% were observed.

**TABLE 3:** OD measurements (OD570 ± SD) and relative biofilm biomass (%) of *Escherichia coli* isolates following treatment with the ethanolic extract of *Physalis alkekengi* .

Concentration (mg/mL)	EC1 (Mean ± SD, % vs. control)	EC2 (Mean ± SD, % vs. control)	EC3 (Mean ± SD, % vs. control)
10	0.345 ± 0.002 (100.0%)	0.635 ± 0.245 (100.0%)	1.351 ± 1.18 (100.0%)
5	0.554 ± 0.207 (160.5%)	0.667 ± 0.23 (105.0%)	0.602 ± 0.255 (44.6%)
2.5	0.565 ± 0.202 (163.7%)	0.578 ± 0.134 (91.1%)	1.052 ± 1.028 (77.9%)
1.25	0.399 ± 0.192 (115.5%)	0.465 ± 0.087 (73.3%)	1.073 ± 0.415 (79.4%)

*Note:* Biomass values were normalised to the untreated growth control (*E* = 100%).

In comparison, the aqueous extract was less effective. At 10 mg/mL, it reduced biofilm biomass by around 34% in EC1 and 55% in EC2. For EC3, however, minimal inhibition was detected, with biofilm biomass remaining near 70% of the control. At lower concentrations (5 and 1.25 mg/mL), the aqueous extract produced no significant reduction in most strains. Collectively, these findings indicate that the ethanolic extract had stronger and more consistent antibiofilm effects, whereas the aqueous extract showed its most notable activity only in EC2.

**FIGURE 6:**
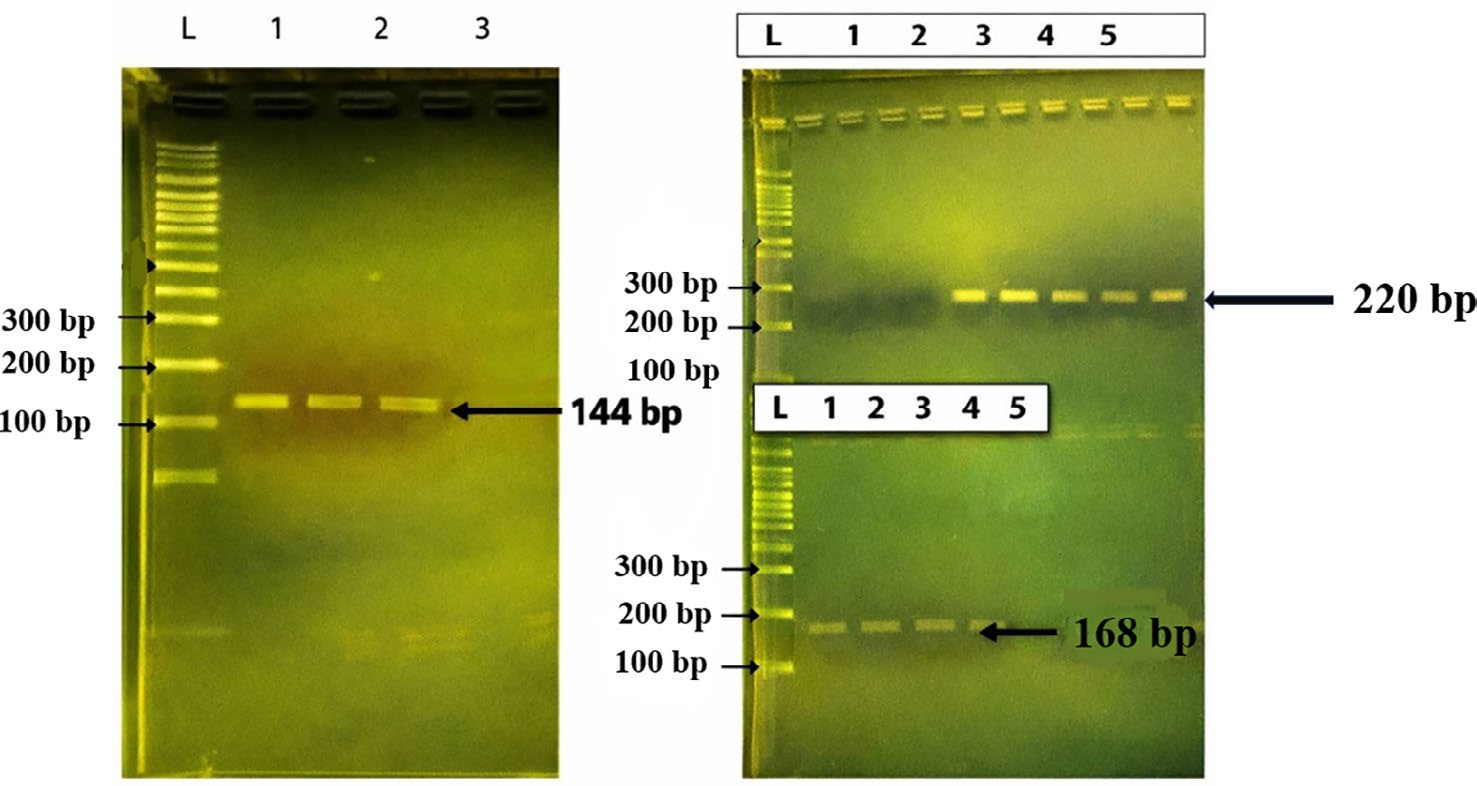
PCR amplification patterns of target genes from *Escherichia coli* strain EC2 analysed by agarose gel electrophoresis. (A) Amplification of the *16S rRNA* gene (144 bp) at three annealing temperatures (lanes 1–3). (B) Amplification of *luxS* (220 bp) and *fimH* (168 bp) genes at five different annealing temperatures (lanes 1–5). Lane L indicates the 100 bp DNA ladder.

Phenotypic identification of EC2 as *E. coli* was initially established through routine biochemical and differential tests. Molecular confirmation using PCR amplified single, specific products corresponding to *16S rRNA* (144 bp), *luxS* (220 bp) and *fimH* (168 bp) without nonspecific bands (Figure 6). The presence of quorum‐sensing and adhesion‐associated genes supported further expression analyses.

**FIGURE 7:**
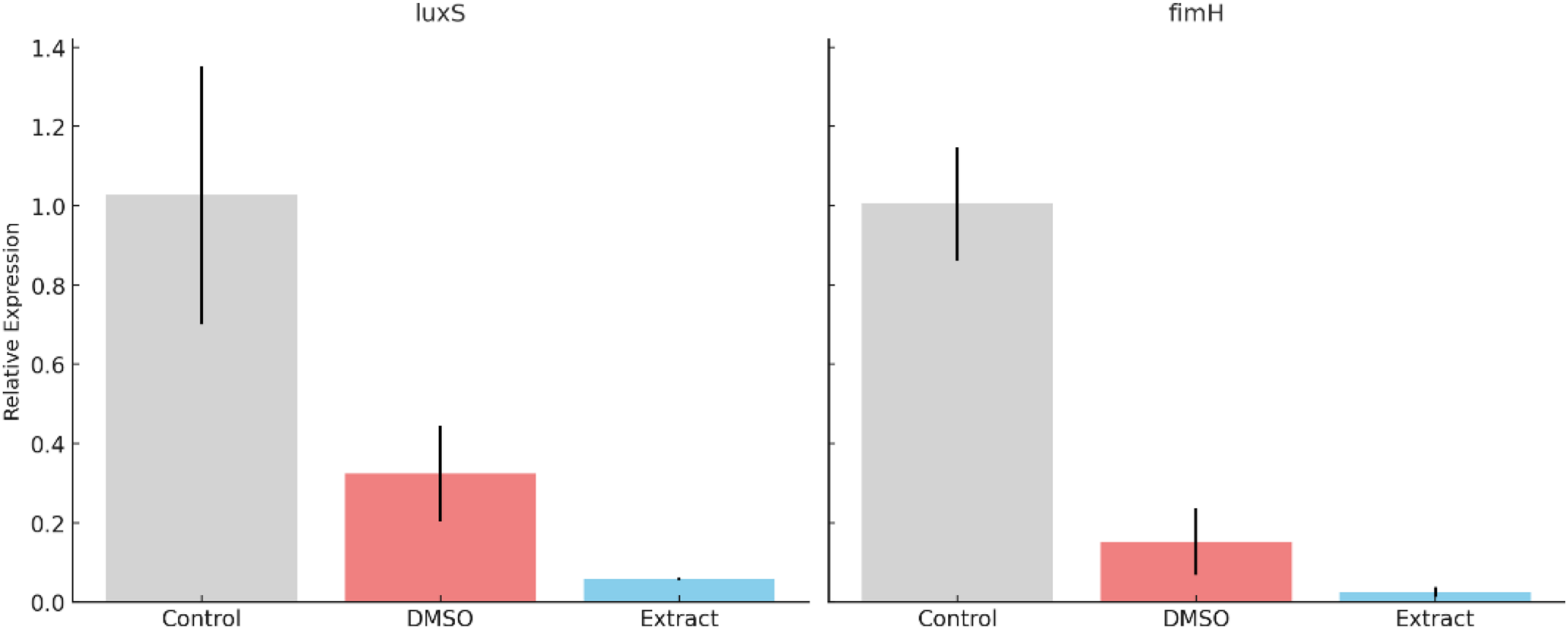
Relative transcription levels of *luxS* and *fimH* in EC2 after treatment with the ethanolic extract of *Physalis alkekengi* . Results (mean ± SD, *n* = 3) indicate significant downregulation of both targets compared with the DMSO control (*p* < 0.05).

qPCR demonstrated that exposure of EC2 to the ethanolic extract significantly reduced transcription of both *luxS* and *fimH* compared to the DMSO control (*p* < 0.05). Relative *luxS* expression dropped to 0.0586 ± 0.0043 in treated samples versus 0.3236 ± 0.1207 in controls (Figure 7). At the highest concentration tested (10 mg/mL), a 63% reduction in *luxS* expression was observed, while reductions at 5 and 1.25 mg/mL were 46% and 48%, respectively. Similarly, *fimH* expression decreased to 0.0249 ± 0.120 compared to 0.1525 ± 0.0839 in the control. These transcriptional alterations indicate that the ethanolic extract of *P. alkekengi* not only inhibits growth and biofilm formation but also suppresses key genes involved in QS and adhesion.

## 4 Discussion

The increasing prevalence of MDR *E. coli* represents a major challenge to global public health and highlights the urgent need for alternative antimicrobial strategies. Plant‐derived bioactive compounds have attracted growing attention as potential sources of antimicrobial and antivirulence agents. In the present study, extracts of *P. alkekengi* demonstrated noticeable inhibitory activity against clinical isolates of *E. coli* , particularly when ethanol was used as the extraction solvent. These findings suggest that phytochemical constituents of this medicinal plant may contribute to suppressing bacterial growth and virulence‐related behaviors.

Phytochemical analysis revealed clear differences between the aqueous and ethanolic extracts. The ethanolic extract contained higher levels of flavonoids and flavonols, whereas the aqueous extract exhibited a relatively greater concentration of total phenolic compounds. Variations in the abundance of these metabolites can be influenced by extraction solvent polarity, plant growth conditions and environmental stress factors that affect secondary metabolite biosynthesis (Zuberi et al. 2017; Wang et al. 2023). The higher antimicrobial activity observed in the ethanolic extract may therefore be attributed to the enhanced solubility and recovery of specific flavonoid compounds known to possess antibacterial properties (Smith et al. 2019; Jones et al. 2018).

In antimicrobial susceptibility assays, the ethanolic extract showed stronger inhibitory activity than the aqueous extract, achieving complete growth inhibition of the EC3 isolate at 10 mg/mL. Such differences in susceptibility among isolates are not unexpected, as clinical *E. coli* strains often exhibit substantial genetic variability and diverse resistance mechanisms (Ballén et al. 2022). The absence of clear bactericidal activity in some assays suggests that the extracts may act primarily through bacteriostatic mechanisms, slowing bacterial proliferation rather than directly causing cell death. This observation is consistent with previous reports indicating that many plant‐derived compounds interfere with essential metabolic or regulatory pathways without immediately disrupting cellular integrity (Wiegand et al. 2008; Alshammari et al. 2023).

Results obtained from agar well diffusion assays were generally consistent with those from broth‐based experiments, although some variability in inhibition zones was observed. Differences between these methods can arise from limitations in the diffusion of compounds through solid media and variations in bacterial growth dynamics across different experimental conditions (Wang et al. 2021). Moreover, factors such as outer membrane permeability, efflux pump activity and the metabolic state of bacterial cells may influence their susceptibility to phytochemical compounds (Ballén et al. 2022).

An important aspect of this study is the demonstrated ability of the ethanolic extract to significantly inhibit biofilm formation. Biofilm development plays a crucial role in the persistence and pathogenicity of *E. coli* , particularly in UTIs, where bacterial adhesion and community formation facilitate long‐term colonisation. The reduction in biofilm formation observed in this study was accompanied by decreased expression of the *luxS* and *fimH* genes. The *luxS* gene participates in the synthesis of autoinducer‐2 (AI‐2), a signalling molecule involved in QS and intercellular communication among bacteria. Meanwhile, *fimH* encodes the adhesive tip protein of type 1 fimbriae, which is essential for bacterial attachment to host epithelial cells (Klemm and Schembri 2000; Sheikh et al. 2001). The downregulation of these genes suggests that *P. alkekengi* extracts may interfere with regulatory networks governing adhesion and QS.

The ability of plant‐derived compounds to disrupt QS pathways has been reported in several medicinal plants and is considered a promising antivirulence strategy. Instead of exerting strong selective pressure that leads to antibiotic resistance, antivirulence approaches aim to weaken bacterial pathogenicity and reduce infection severity. Similar QS‐inhibitory effects have been reported for extracts of *Eucalyptus globulus* and *Lippia origanoides*, which have been shown to suppress biofilm formation and virulence gene expression in pathogenic bacteria (Alshammari et al. 2021; Martínez et al. 2023; Crescenzi et al. 2023).

From an environmental microbiology perspective, interference with QS systems may also influence microbial interactions beyond clinical settings. QS regulates numerous ecological behaviours, including biofilm formation, nutrient acquisition and microbial competition in natural environments. Therefore, plant‐derived metabolites capable of modulating these signalling systems could potentially affect microbial community dynamics in diverse ecological niches (Kalia 2013). Understanding such interactions is particularly relevant given the increasing interest in natural products as environmentally compatible antimicrobial agents.

Overall, this study's results highlight the promising antimicrobial and antivirulence potential of *P. alkekengi* extracts against *E. coli* . The ethanolic extract, in particular, demonstrated notable activity in inhibiting bacterial growth, reducing biofilm formation and downregulating key virulence‐associated genes. Further investigations are needed to isolate and characterise the specific bioactive compounds responsible for these effects and to clarify their molecular mechanisms of action. Such studies may contribute to the development of novel therapeutic or preventive strategies targeting bacterial communication and pathogenicity.

## 5 Conclusion

The ethanolic extract of *P. alkekengi* exhibited consistent antibacterial and antibiofilm activity against pathogenic *E. coli* , underscoring its potential as a source of bioactive compounds with clinically relevant effects. In addition to limiting bacterial proliferation, the extract markedly decreased transcription of the QS gene luxS and the adhesion‐associated gene fimH, suggesting a broader antivirulence effect that targets both cell–cell communication and early colonisation events. Taken together, these properties identify *P. alkekengi* as a promising natural reservoir for developing approaches that weaken infection establishment and biofilm‐associated tolerance to treatment.

Beyond implications for clinical management, the ability of *P. alkekengi* metabolites to modulate QS and adhesion‐related pathways suggests that such compounds may also influence microbial signalling, surface colonisation and persistence in environmental niches. This raises the possibility that plant‐derived QS modulators contribute to shaping microbial community structure and function outside the host, linking traditional medicinal plants to wider ecological processes.

Building on the present work, subsequent studies should aim to isolate and structurally characterise the active phytochemical constituents and to clarify their molecular targets within bacterial regulatory networks. In parallel, appropriately designed in vivo models will be required to evaluate efficacy, pharmacokinetics and safety. Such efforts will be crucial to determine whether *P. alkekengi* ‐derived compounds can be translated into practical interventions against antibiotic‐resistant *E. coli* infections and persistent, biofilm‐driven diseases.

## Author Contributions

**Mansoureh Esfandiyari Doolaby:** writing – original draft, methodology, investigation. **Akbar Norastehnia:** investigation, methodology, writing – review and editing, writing – original draft. **Mohammad Javad Mehdipour Moghaddam:** conceptualization, supervision, project administration, writing – review and editing, writing – original draft, validation, investigation, methodology.

## Ethics Statement

The study was conducted in accordance with the Declaration of Helsinki, and the protocol was approved by the Ethics Committee of the University of Guilan, Rasht, Iran (ID: IR.GUILAN.REC.1405.018) on 04/13/2026.

## Conflicts of Interest

The authors declare no conflicts of interest.

## Data Availability

The data that support the findings of this study are available on request from the corresponding author. The data are not publicly available due to privacy or ethical restrictions.
